# Supplementary material for: Are carbon emissions trading and green financial instruments synergistic? -Comprehensive quantitative research based on content analysis
Source: PLoS One. 2024 Mar 7;19(3):e0298601. doi: 10.1371/journal.pone.0298601 (PMC10919601; doi:10.1371/journal.pone.0298601)
Supplement: S1 Data — (ZIP) [file pone.0298601.s001.zip › Data/地级市Z_F工作报告.zip!/╣π╢1⁄2/╒╪╟∞╩╨/└┤╫╘╣1⁄2╓┌║┼í╛┬φ┐╦╩2╛▌═°í┐_2006.docx]

各位代表：

　　我代表市人民政府向大会作政府工作报告，请予审议，并请政协各位委员和列席人员提出意见。

　　一、二○○五年工作取得实效，“十五”计划圆满完成

　　2005年，在中共广东省委、省人民政府和中共肇庆市委的正确领导下，在市人民代表大会及其常委会和市政协的监督支持下，市政府坚持以科学发展观统领经济社会发展全局，深入贯彻省委、省政府各项部署和张德江书记视察肇庆重要讲话精神，认真落实市第九次党代会和市委九届四次全会的工作部署，做到“谋全局、促发展、重为民、抓落实”，抢抓机遇，团结拼搏，克服宏观经济环境偏紧、自然灾害严重等困难，全力推动经济社会快速健康协调发展。全市经济保持近年来形成的良好发展势头，主要经济指标快速增长，达到或超过预期目标，社会各项事业取得新发展。

　　（一）国民经济呈现快速发展的良好态势.

　　经济增长速度再创新高。实现肇庆市生产总值453.55亿元（经全国经济普查核准，2004年肇庆市生产总值为390.56亿元），比上年增长14.3%（按可比价计算，下同），高于预期目标和全省平均增幅，成为“十五”时期最高增速，并创8年来新高。一、二、三产业增加值分别比上年增长5.8%、22%和16.1%。

　　工业经济发展速度加快。坚定不移地推进工业立市，重点推动电子信息、生物制药、机械制造、金属加工、汽车配件、林产化工、林浆纸一体化等产业向集群化方向发展。着力打造工业发展载体，工业园区和产业集聚基地发展到25个，进园企业702户。肇庆高新区加快规划和建设，与省国资委签订了共同开发园区的框架性协议。稳步推进市属国企改革，完成了星湖科技公司股权分置改革，实施市属国企产权改革方案67个，共筹措和支付改革资金7119万元，安置职工2993人，盘活资产4.38亿元。狠抓技术改造，一批企业通过技改扩产规模迅速扩大。有4个产品被评为广东省名牌产品。全部工业总产值达388.81亿元，比上年增长23.2%；规模以上工业增加值比上年增长25.4%，超过预期目标和全省平均增幅，在全省排第六位。工业用电量比上年增长25%。

　　招商引资成效显著。全面落实招商引资工作责任制，重新组建市招商局，加大财政投入。成功举办第三届“肇庆金秋”经贸洽谈会，签约合同项目209宗，合同投资总额245亿元。组织赴台湾和赴顺德、南海、东莞等地的大型招商活动，参加省组织赴美国、澳大利亚等国的经贸洽谈活动，均取得明显效果。新批注册合同外资额8.77亿美元，比上年增长58.5%；实际吸收外资6.03亿美元，比上年增长25%，超过预期目标，总量稳居全省山区市首位。

　　重大项目谋划建设取得重大突破。把谋划建设重大项目作为实现跨越发展的战略举措，精心组织，扎实推进。首期投入42亿元的亚洲铝业大旺工业城于去年11月正式试投产，我市有史以来最大的投资项目、总投资达78.9亿元的广贺高速公路三水至怀集段正式动工建设。投资额9.5亿元的肇庆城东新区基础设施项目全面动工建设。肇庆至广州新机场快速干线、肇庆内河核电项目等正在抓紧筹划。

　　固定资产投资持续增长。交通、能源、水利基础设施和城市建设加快推进。肇庆大桥南引道扩建、西江大桥扩建工程已建成通车，321国道文明样板路改造正式启动，县通镇公路上等级改造工程已经完成，实现了县到镇基本通三级以上公路的目标，改造建设镇通村硬底化道路845.8公里，累计完成总工程任务的94.5%。西江航道综合整治和肇庆新港等交通项目、景丰联围加固达标等水利工程、大旺220千伏等一批输变电工程正在抓紧建设。肇庆城区宋城路改造基本完成，端州七、八路改造等工程正在推进，“美化、绿化、亮化”工程建设也取得新进展。全社会固定资产投资完成177.86亿元，比上年增长22.9%。

　　农业生产和县域经济取得新发展。认真落实中央1号文件精神，加强农业基础设施建设和信息化建设，战胜了严重春旱和百年一遇特大洪涝灾害，确保农业生产大灾之年不减产。粮食播种面积比上年增加2.8万亩，农业总产值达202.19亿元，比上年增长8.1%。大力推进农业产业化和农业基地建设，十大特色农业基地和十大农业品牌规模扩大，知名度进一步提高。有3个农产品被评为省级名牌。各类农业产业化组织发展到161个，80家农业龙头企业销售收入41.3亿元，带动农户达25.9万户。坚持以工业化带动农村“三化”，突出以资源和产业招商，引进建设一批资源型工业项目，初步形成各具特色的县域工业产业集群，4个山区县规模以上工业增加值增幅超过全市平均水平。以县域城区为重点，中心镇规划建设管理得到加强，打造了一批特色产业强镇。

　　第三产业发展态势良好。制定旅游发展总体规划，出台促进旅游发展政策文件，推进旅游资源整合，丰富了千里旅游走廊等品牌内涵，打造了封开国家地质公园、广宁竹海国家森林公园、怀集燕峰温泉等一批特色鲜明的景区景点。怀集、封开、德庆先后引入南湖国旅、广之旅等知名企业参与旅游投资开发和策划推介，促进旅游产品转型升级。城市住宿设施接待旅游者人数512.45万人次，旅游收入41.27亿元，分别比上年增长8.2%和17.2%。房地产业保持健康发展态势，完成房地产开发投资21.3亿元，比上年增长21.8%，一批规模大、档次高的房地产项目和星级酒店已动工建设。外贸出口14.16亿美元，比上年增长14.2%，超过预期目标。加强物流产业规划研究，优化物流业发展环境，一批知名物流企业相继登陆城区，商贸、餐饮业发展水平进一步提升，消费日趋兴旺，社会消费品零售总额142.29亿元，比上年增长17.7%。

　　民营经济进一步发展壮大。多次召开会议专题研究民营经济工作，制定实施了鼓励外来投资和民营经济发展、稳定外来工等一系列政策措施，加快中小企业信用担保体系和社会化服务体系建设，成立“肇庆市中小企业融资辅导中心”，扶持设立3家信用担保公司，优化民营经济发展环境，促进了民营经济发展。规模以上民营工业总产值比上年增长45%，占全市规模以上工业总产值的32.9%；个体私营企业实现税收6亿元，比上年增长21%。

　　财政收入增幅加大。加快公共财政体系建设，全面推行部门预算改革，扩大国库集中支付试点，首次将市直政府性资源收益纳入政府统一管理，实行了市直机关公务员统一岗位津贴和机关事业单位统一公用经费标准的改革，初步建立起财政支出绩效评价制度。加强财税征管，地方财政一般预算收入20.43亿元，按市可比口径比上年增长21.5%（按省可比口径，即包含农业税因素计算，比上年增长19%），比GDP增幅高出7.2个百分点。国、地税系统税收收入分别比上年增长28%和20.6%。

　 人民生活水平得到提高。随着经济的发展，城乡居民生活不断改善。城镇居民人均可支配收入10097元，比上年增长9%；农村居民人均纯收入4558元，比上年增长5%；年末城乡居民储蓄存款余额335.93亿元，比年初增长14.6%。城乡消费结构逐步升级，居住条件不断改善。

　　（二）科技、教育、文化等社会各项事业全面进步

　　科技兴市迈出新步。顺利通过全国科技进步考核，实施省级以上各类科技计划73项，获得省级以上科学技术奖7项，其中国家科学技术二等奖1项，省科学技术一等奖1项。与中山大学、中国科学院华南植物园等院校开展了多项合作。目前，已拥有省级以上高新技术企业69家、民营科技企业67家、农业科技创新中心3家、专业镇8个，成为全省山区地级市专业镇建设先进地区之一。成功举办首次科学技术贸易洽谈会。信息化取得明显进展，以有线电视为载体的农村科技信息“直通车”全面开通。大力实施人才工作基础工程、素质提升工程和引智纳贤工程，继续委托中山大学举办领导干部培训班、选派年轻干部赴英国进修，共引进硕士研究生以上学历人才122人、本科学历人才1615人，分别比上年增长103%和101%。

　　教育强市扎实推进。继续加大财政对教育的投入，中小学布局调整取得重大进展，累计已完成504所中小学校改造建设。引进和培养了大批高素质教师。九年义务教育进一步巩固提高，各项指标均居全省中上水平。普通高中发展加快，素质教育不断推进，课程改革全面铺开，高考取得历史最好成绩。职业教育和民办教育蓬勃发展，我市成为广东省首个职业技术教育培训基地。高等教育规模扩大，发展水平提高。

　　文化名市建设加快。成功举办国家历史文化名城2005年年会暨第12次学术研讨会，发表了旨在保护历史文化名城的《肇庆宣言》。加强历史文化资源的挖掘整理、保护和开发利用，对22条古村落进行挖掘、开发、建设，完成“肇庆府城”等保护规划，编辑出版了肇庆历史文化丛书。广场文化呈现蓬勃生机，打造了一批文化节庆品牌。农村广播电视网络“村村通”工程稳步推进。开展文化体制改革，文化市场管理有序，文化产业健康发展。

　　生态环境保护得到加强。投入3000万元整治星湖环境，水质保护初见成效。整体环境质量继续保持优良，森林覆盖率达65.1%，城区生活污水处理率达70%以上，生活垃圾无害化处理率达100%；创建国家环保模范城市考核指标全部达标，通过了国家环保总局考核验收组的考核验收。

　　卫生、体育等事业取得新发展。公共卫生体系建设不断完善，市、县疾控中心建设加快推进，有效防控了非典、禽流感等重大传染病疫情。农村薄弱卫生院改造取得成效，建成广东省卫生村39条。全民健身活动广泛开展，体育设施有较大改善，我市运动员在第十届全运会上夺得13枚金牌，居全省第二。经济普查与人口抽查进展顺利。人口和计划生育工作达到目标要求。外事、侨务工作取得新突破，与英国波尔顿市结为友好城市。国防后备力量、国防动员和人民防空建设得到加强，“双拥”工作扎实有效，部队和民兵预备役在抢险救灾和经济建设中发挥了积极作用。殡葬改革得到落实。审计、妇女儿童、民族、宗教、地方志、新闻出版、质量监督、气象、人防、档案、老龄、残疾人、社会救助等工作取得新进展。

　　（三）民主法制和精神文明建设不断加强

　　民主法制建设不断完善。坚持向人民代表大会及其常委会报告工作、向人民政协通报情况，认真办理各类建议、议案、提案。向市人大常委会提交各类专项工作报告20件，组织办理人民代表建议121件，政协提案、建议177件。广泛听取各方面意见，重大事项社会公示和听证制度得到落实。“四五”普法工作通过省的检查验收并被评为“优秀”。贯彻实施国务院《全面推进依法行政实施纲要》，组织公务员学习《行政许可法》和《公务员法》等法规，建立了实施行政许可相关配套制度。开展“规范执法行为，促进执法公正”专项整治。顺利完成村级换届选举工作，完成省下达的创建民主法治示范村任务，2条村被评为全国民主法治示范村。积极做好接访工作，接访办结率和停访息诉率均居全省前列。

　　维护稳定工作扎实有效。打击“法轮功”邪教组织和矛盾纠纷排查调处工作得到加强。调处各类矛盾纠纷4059宗，成功率达97.4%；群体性事件比上年下降45%，没有发生打砸抢烧等影响恶劣的群体性事件。深入开展“严打”整治行动，侦破了一批大要案及黑恶案件。加强治安防控体系建设，县(市)公安局完成110、119、122三台合一。乡镇、街道全部设立了维稳及综治机构；中小学全部聘任了兼职法制副校长。共创建安全文明小区1990个。我市被授予“全国社会治安综合治理优秀地市”称号，在全省维稳及综治工作检查考核中被评为优秀。安全生产形势保持稳定。

　　精神文明建设不断推进。“爱国、守法、诚信、知礼”现代公民教育活动深入开展，“开放兼容、务实进取”的现代肇庆人精神得到弘扬。认真部署创建全国文明城市工作。“创建学习型组织，争当知识型员工”活动在机关、企事业单位蓬勃开展，掀起学习贾东亮艰苦创业先进典型活动。文明行业、文明单位、文明社区和民营企业文明创建活动扎实推进。生态文明村建设走在全省前列，已建成生态文明村700多条。

　　（四）贯彻为民务实要求取得明显成效

　　“十项民心工程”取得新进展。免征农业税、取消和降低涉农收费减轻农民负担3137万元。高度重视遭受特大洪水群众生产生活问题，市财政投入965.4万元帮助灾区群众重建家园、恢复生产。各级免收农村义务教育阶段书杂费人数44042人，其中省政策性免收28871人。在肇庆城区规划建设500套安居住房，市财政已预支1233.6万元征地款，征地128亩。全市投入资金8483万元，解决农村18.3万人饮水难工程基本完成。多渠道筹集5397.11万元投入农村改水改厕，新增自来水受益人口10.6万人，增加使用无害化厕所农户4.29万户。各级投入5170.5万元完成2760户农村贫困户危房改造，新建面积25.6万平方米，受益人口1.38万人。已有130多万人参加新型农村合作医疗。积极追收清欠工程款8.88亿元，其中属政府工程欠款5.87亿元、欠农民工工资3390万元已全部偿还。在全省率先全部清欠农民征地补偿款，共9144万元。

　　就业和社会保障工作得到加强。落实再就业小额担保贷款、农村青年技能培训等扶持措施，新增就业岗位31890个，下岗失业人员再就业5475人，“4050”人员再就业1915人，农村劳动力转移就业83624人，年末城镇登记失业率为2.7%。全面完成社会保险扩面和征缴任务，征缴社会保险费8.4亿元，征缴率达99.4%，支付各项社会保险待遇7.3亿元，支付率保持100%，实现社保基金有所结余。认真解决城乡困难群众低保问题，向6.29万低保对象发放低保金2476万元。开通了“肇庆慈善网”。

　　政府服务不断改进。完善经济工作责任制考核办法，制定落实了政府行政审批制度、部门行政首长问责制度、政府重大投资项目报告制度、政府性固定资产投资管理制度等，促进行政服务规范化。扩大了政府公共资源配置市场化范围。在执行省政府下放审批权项目基础上，把市一级政府80多项审批权和事权下放给各县（市）区。加强调查研究，深入解剖城区报建环节的典型，提出整改意见和措施，促进了行政效率提高和投资环境改善。

　　廉政建设深入推进。认真学习和贯彻落实中央惩治和预防腐败体系《实施纲要》和省委《实施意见》。围绕农村征用土地、城镇房屋拆迁、企业重组改制和破产中损害群众利益，以及拖欠农民工工资、企业违法排污等群众反映的热点问题进行专项治理；深入开展民主评议政风行风，开办“行风热线”节目，组织11个政府部门负责人在电视台、电台与市民直接沟通，有效处理举报投诉322宗；深化行政审批制度改革，推进政务公开，加强行政效能监察，严肃查处了违反行政纪律的行为。

　　各位代表，我们通过去年的工作，不仅加快了经济社会发展，实现“三年见成效”的目标，而且促进了“十五”时期目标任务的圆满完成。“十五”期间，肇庆市生产总值实际年均增长11.5%，超过计划目标0.5个百分点；其中第二产业增加值实际年均增长16.1%，超过计划目标3.8个百分点；全社会固定资产投资实际年均增长15.7%，超过计划目标4.2个百分点；外贸出口实际年均增长13.9%，超过计划目标3.9个百分点。人口控制等各项社会发展指标也达到计划要求。特别是市第九次党代会以来，我市经济社会发展速度明显加快，呈现不少新亮点：一是经济增长速度不断创新高，肇庆市生产总值年均增长速度近三年比头两年提高3.8个百分点；二是工业经济加速发展，工业总产值年均增长速度近三年为18.7%，远高于头两年10.4%的水平。2003年以来共引进投资1000万元以上工业项目525项，目前在建投资超过5000万元的100多项，预计达标达产后可增加工业总产值600亿元；三是招商引资成效显著，“十五”时期实际吸收外资达27.5亿美元。三届“肇庆金秋”经贸洽谈会共签订项目合同投资额1063亿元；四是固定资产投资大幅增长，“十五”时期累计完成投资623亿元，超过“八五”、“九五”时期的总和，其中近三年投资达437.32亿元；五是财政实力增强，地方财政一般预算收入年均增长速度近三年比头两年提高6.2个百分点，去年首次超过20亿元；六是随着经济发展，人民生活水平相应提高。城镇居民人均可支配收入从2002年7335元增加到去年的10097元，农村居民人均纯收入从2002年3968元增加到去年的4558元。与此同时，“十五”期间，我市交通、能源、水利基础设施建设和城市建设取得重大突破，体制改革和社会保障建设深入推进，科技、教育、文化、卫生、体育事业蓬勃发展，民主法制建设、维稳工作和精神文明建设不断加强。

　　上述成绩的取得，是省委、省政府和市委正确领导的结果，是各级人民代表大会及其常委会和人民政协监督支持的结果，是全市人民团结奋斗、求真务实、开拓进取的结果，也是社会各界关心支持的结果。在此，我代表市人民政府，向全市工人、农民、干部职工、外来务工人员、驻肇单位、驻肇部队、武警官兵以及各界人士，致以崇高的敬意！向关心和支持我市建设的外来投资者、港澳台同胞、海外侨胞和国际友人表示衷心的感谢！

　　我们一年来的工作虽然取得了很大成效，但仍存在不少困难和问题：经济总量和规模仍然偏小；自主创新能力不足，具有较强竞争力和辐射带动能力的优势产业、支柱企业较少；体制性障碍仍未完全消除，市直重点国有控股企业历史遗留的深层次问题逐渐显露；招商引资项目履约率和资金到位率有待进一步提高，有的重点项目建设进度达不到计划要求；区域经济发展不平衡等问题仍较突出，部分下岗职工和农村群众生活还比较困难；城市规划建设管理水平仍有待提高；政府职能部门及其工作人员的工作作风和服务水平还有待改进，等等。这些都需要我们高度关注，采取切实有效措施认真解决。

　　二、科学规划“十一五”时期工作，努力实现跨越发展目标

　　根据市委九届五次全会通过的《中共肇庆市委关于制定全市国民经济和社会发展第十一个五年规划的建议》，我们组织编制了《肇庆市国民经济和社会发展第十一个五年规划纲要（草案）》（以下简称《规划纲要》），提交本次大会审议。在规划编制之前，首先多方面开展前期研究工作，委托中山大学专家开展肇庆市产业选择和发展研究，先后组织开展了城市规划、旅游发展、中心城区商业网点布局、物流业发展、县域经济发展等专题规划的研究工作。2004年下半年，由市发改局召开“十五”计划执行情况评估会议，在总结分析“十五”发展情况的基础上，提出《肇庆市国民经济和社会发展“十一五”规划基本思路》，明确职责分工，加强调查研究，从2005年初开始着手总体规划和专项规划的编制工作。经过一年多努力，完成了《规划纲要》和10多个专项规划的初稿，然后按照市委九届五次全会精神及市委“十一五”规划《建议》的部署和要求进行认真修改、完善，并广泛征求有关专家、学者和市经济技术顾问团的意见，听取了市政协常委、部分政协委员和民主党派、无党派人士、工商联负责人的意见建议，书面征求各县（市）区政府、市直有关部门和市人大代表的意见，市政府召开了常务会议讨论研究，并提交市委常委会审议。《规划纲要》总体贯彻市委的决策部署和市委九届五次全会精神，体现政府工作抓落实的要求，是充分发扬民主、集中各方智慧、集体研究决策的成果。《规划纲要》经本次大会审议通过后，将正式颁布实施。

　　“十一五”时期是我市加快发展、跨越发展、协调发展的关键时期，也是重要的机遇期，国内国际形势总体有利于我们加快发展。经过改革开放20多年的努力，我市产业基础不断夯实，发展环境不断改善，特别是市第九次党代会以来，经济的快速发展为我市实现跨越发展打下了坚实的基础。我们要认真贯彻落实市委九届五次全会精神，认清形势，统一思想，明确任务，坚定信心，抢抓机遇，乘势而上，努力开创改革开放和现代化建设新局面。

　　“十一五”时期我市经济社会发展的指导思想是：坚持以邓小平理论和“三个代表”重要思想为指导，以科学发展观统领经济社会发展全局，全面贯彻党的十六届五中全会和省委九届七次、八次全会精神，坚持落实市第九次党代会以来各项工作部署和市委九届五次全会精神，按照“五个统筹”和“三个最适宜”的要求，以建设繁荣活力、文明法治、和谐安康肇庆为目标，坚持以人为本，狠抓发展第一要务，优化经济结构，培育支柱产业，转变增长方式，增强创新能力，壮大经济总量，提高发展质量，促进经济社会加快发展、跨越发展、协调发展和可持续发展，增强经济实力与综合竞争力，提升人民生活水平，为全面建设小康社会、推进社会主义现代化打下更坚实的基础。

　　“十一五”时期全市经济社会发展目标是：肇庆市生产总值年均增长13%以上，其中一、二、三产业年均增长5%、21%和13%左右，GDP总量到2007年比2002年接近翻一番，实现“五年大发展”的宏伟目标；到2010年比2005年再接近翻一番。全社会固定资产投资年均增长18%以上。社会消费品零售总额年均增长13%以上。万元GDP能源消耗比“十五”期末降低13%以上。经济增长质量和效益明显提高，经济综合实力明显增强。初步建成花园式、生态型、现代化大城市基本框架。同时，区域协调发展创新局面，经济体制及市场体系进一步完善，文化教育科技等各项事业全面发展，社会文明程度明显提高，生态环境进一步优化，社会保障体系更加健全，社会更加稳定和谐，人民生活总体达到宽裕型小康水平，繁荣活力、文明法治、和谐安康肇庆建设迈上新台阶。

　　“十一五”时期着力实施“五大战略”。一是工业主导战略，坚持把工业放在牵动全局的战略地位，提高工业规模化、集约化发展程度。二是重大项目带动战略，把重大项目作为实现肇庆跨越发展的重要支撑，力争一批项目列入国家和省的“十一五”专项规划。三是东引西连战略，加速与广佛经济圈对接融合，加强泛珠三角区域合作发展。四是科教兴市和人才强市战略，充分发挥教育、科技、文化、人才对经济社会发展的智力支撑作用。五是区域协调发展战略，加强分类指导，优化资源配置，实施梯度推进，促进三大经济板块同步发展。

　　（一）壮大优化工业经济，推动工业率先实现跨越发展。坚持优先发展工业，走科技含量高、经济效益好、资源消耗少、人力资源得到充分发挥的新型工业化道路。加快工业基地建设，大力培育支柱产业，积极发展龙头企业，努力扩大工业规模。发展壮大电子信息和装备制造、汽车零配件、金属加工、生物医药、林浆纸一体化等优势产业，培育发展新能源、再生资源加工等新产业，提升发展林产化工、新型建材、食品饮料等传统产业，打造一批具有区域竞争优势的产业集群。大力推进技术改造，努力创造知名品牌，做大做强一批支柱企业，做精做专一批中小企业。力争今后五年工业总产值年均增长22%，到2010年达到1000亿元左右，成为广东省重要的制造业基地和资源型产业基地。

　　（二）大力发展现代农业，努力建设社会主义新农村。按照“生产发展、生活宽裕、乡风文明、村容整洁、管理民主”的要求，坚持增加农民收入为目标，努力解决“三农”问题。强化以水利设施为重点的农业基础设施建设。推进农业产业化、基地化和现代化。发展品牌农业、效益农业、特色农业和生态农业，建设高效林业。加快农业科技进步和农业信息化、标准化、机械化建设，完善农业服务体系。加强农村规划建设管理，全面开展创建生态文明村活动，积极发展农村公共事业和农村教育、卫生、文化、社会保障等社会事业，稳步推进社会主义新农村建设。

　　（三）加快发展服务业，提升第三产业现代化水平。推进以中心城区为龙头的千里旅游走廊建设，谋划建设大型旅游项目，规划开发羚羊峡、烂柯山等景区，努力建设成为国内重要的观光、休闲、度假、会议旅游目的地城市。加强服务标准体系建设，积极培育、引进为生产服务的物流产业，扶持发展物流龙头企业和港口物流基地，建成珠江流域重要物流中心。加强房地产开发规划，提高房地产业发展水平。建设区域性服务中心，加快发展金融、保险、会展、商务、中介、咨询、医疗等现代服务业。

　　（四）全力推进招商引资，发展壮大民营经济。加大招商引资力度，创新招商机制，优化招商结构，强化招商服务，着力引进大企业、大项目。优化出口结构，扩大出口规模，提高质量效益，力争外贸出口年均增长12%。落实扶持民营经济政策，吸纳珠三角发达地区民营资本和壮大本地民营企业并重，推动民营资本与高新技术产业、与改造传统优势产业、与深化公有企业改革对接，促进民营经济发展迈上新台阶。

　　（五）积极谋划建设重大项目，增强跨越发展的支撑力。继续谋划一批对实现经济社会跨越发展起支撑作用的重大项目。规划重点建设项目137项，“十一五”时期投资约700亿元。积极运用市场化等手段，加快交通、能源、通讯和城市化项目建设，确保项目建设进度和工程质量。

　　（六）打造三大经济板块，统筹区域经济协调发展。着力打造东南部工业经济板块，积极引进大（大项目）、新（高新技术）、密（资金密集型）产业。推进以肇庆高新区为龙头的园区集约开发建设，力争高新区成为国家级高新区。做大做强金属加工、汽车配件、再生资源、新型建材等产业集群，努力成为珠三角工业体系的重要组成部分。提升中心城区新经济板块发展水平，发展壮大电子信息、软件开发、生物制药、装备制造等高新技术产业，加快发展旅游、会展、现代物流等城市新经济。积极推进山区县域经济板块建设，坚持走以工业化为龙头、城镇化和农业产业化协调推进的发展路子，重点发展林产化工、林浆纸一体化、矿产品开发利用等资源深加工型产业，加快发展特色旅游、区域性物流和现代种养农业。形成“一核、两带、多极辐射”的区域发展格局。

　　（七）建立自主创新体系，加快转变经济增长方式。深化科技体制改革，依靠科技进步发展循环经济，促进经济增长方式转变。强化企业自主创新能力，推动产学研合作扩大深化，支持重点骨干企业建立技术研究开发中心。推动国民经济和社会信息化，规划建设中巴软件园，以信息化改造传统产业，培育发展软件开发等新兴信息产业，积极发展电子政务和电子商务。力争到2010年，科技进步对经济增长贡献率提高到58%，技术自给率提高到48%。

　　（八）深化体制改革，进一步完善社会主义市场经济体制。创新行政管理体制，推进政企分开、政资分开、政事分开和行政审批制度改革，强化政府社会管理和公共服务职能。构建公共财政体制框架，优化财政支出结构，分类推进事业单位改革。改革投资管理体制，推进政府投资项目咨询评价制、责任追究制和代理建设制。深化国有企业改革，基本完成国有经济战略性调整。加强国有资产监管，强化国有资产绩效评价。健全市场体系，大力发展资本、土地、劳动力、信息、技术等要素市场，完善社会信用体系。

　　（九）建设“一江两岸”大城市，提高城镇化水平。围绕“一江两岸”超百万人口区域中心城市发展战略，修编城市总体规划和专项规划，适时推进行政区划调整，加快构建“山、湖、城、江”和谐相融的“三个最适宜”城市。推进肇庆城区东扩南连，有序推进旧城改造，改善城市面貌。创新城市管理和运营机制，建立国家环保模范城市的长效监督管理机制，保持生态环境良性循环，力争跨入全国文明城市行列。加强中心镇规划和管理，完善城镇公共服务设施。力争2010年城镇化水平达到48%以上。

　　（十）发展科教人才事业，加快建设文化名市。推进教育强市建设，抓好强师振教、双高普九、优质高中、职教基地、高等教育、素质教育等六大工程建设。深化教育体制改革，推进义务教育均衡化、高中教育普及化和高等教育大众化。加快建设科技强市，加大科技基础设施建设投入，大力引进各类科技人才。建立以企业为主体、适应社会主义市场经济、符合科技和产业发展规律的科技创新体系。加快建设文化名市，有效保护和合理利用历史文化遗产，加快文化基础设施建设，推进文化体制改革和创新，开展文化招商，促进文化产业发展。

　　（十一）切实关心人民群众生活，促进社会和谐安康。按照构建民主法治、公平正义、诚信友爱、充满活力、安定有序、人与自然和谐相处的社会主义和谐社会的要求，在努力提高人民群众生活水平的基础上，巩固完善社会保障和公共卫生体系建设，强化公共安全，抓好安全生产，注重食品、药品安全，提高政府保障公共安全和处置突发事件的能力，维护社会稳定。加强精神文明建设，推进民主法治建设，促进社会公平，缩小贫富差距，促进社会和谐安康。

　　（十二）构建资源节约型和环境友好型社会，建设绿色生态山水肇庆。正确处理经济发展与人口、资源、环境的关系。加强人口与计划生育工作，稳定低生育水平。加强耕地和林地保护，实行最严格的土地管理制度。大力节约能源，加强资源综合利用，发展循环经济，提高资源利用率。建立多元化环保投融资机制，加快环境基础设施建设，发展环保型产业。加强环境保护监督。调整林业结构，大力建设公益林，构筑生态屏障体系。严格保护自然生态，推进生态城市建设，积极开展创建绿色城镇、绿色学校、绿色社区、生态示范区等活动。

　　三、着力抓好今年工作，为“十一五”规划顺利实施开好局

　　2006年是实施“十一五”规划开局之年，做好今年工作具有十分重要的意义。我们要以科学发展观统领经济社会发展全局，全面贯彻落实市委九届五次全会精神，按照“抓机遇、谋发展、促和谐、强核心”的工作方针和六个“坚定不移”的要求，扎实抓好各项工作。根据市委“十一五”规划《建议》，结合当前实际，全市经济社会发展预期目标是：肇庆市生产总值增长13%，万元GDP能源消耗降低3%，全部工业总产值增长20.5%，农业总产值增长5.5%，全社会固定资产投资增长20%，社会消费品零售总额增长13.5%，外贸出口增长13%，实际吸收外资增长10%，地方财政一般预算收入增长14%，城镇登记失业率控制在3%以下，人口自然增长率控制在6.9‰以下，城镇居民可支配收入增长8%，农村居民人均纯收入增长4.5%。主要工作措施是：

　　（一）培育发展优势产业，着力壮大工业经济。积极调整工业结构，优化三大经济板块及各板块内部的产业发展布局，加快培育支柱产业，做大做强高新技术产业，培育打造潜在优势产业，改造提升传统优势产业，大力发展矿产资源产业，重点建设金属加工、汽车零部件制造、电子信息装备制造等产业基地，培育特色支柱产业和优势产业集群。抓好重点工业项目和骨干企业的建设和发展，促进亚洲铝业大旺工业城一期工程全面投产，协助鼎丰二期项目立项上马，筹划新建四会金属再生、高要塑料再生等资源再生基地，争取大型电子液晶显示屏等重大工业项目布点我市。积极转变经济增长方式，加快自主创新步伐，以信息化带动工业化，积极开展技术创新，加快企业技术改造，培育打造名牌产品，着力提高企业技术创新能力和竞争能力。加强工业发展载体建设，加紧谋划和推进与珠三角发达地区合作共建产业转移园区，提高各级工业园区和工业集聚基地建设水平。重点打好肇庆高新区作为广东省吸引外资重点园区和山区吸收外资示范园区的品牌，加强与省国资委及省直大型国有企业的合作，加快园区基础设施和配套设施建设，积极申报国家级开发区。市委、市政府决定，在保留高新区一区两园格局不变的前提下，把三榕工业园整体移交端州区管理并实行扩园。端州区要加大开发建设和招商引资工作力度，使三榕工业园成为新的经济增长点和中心城区经济板块的新亮点。

　　（二）全力引进外资民资，提高招商引资质量。继续把招商引资放在经济工作的龙头地位。落实招商引资工作责任制，大力推行项目招商、以商招商、网络招商、中介招商和产业招商。积极开展各类招商活动，加强对欧美、日、韩、台等发达国家和地区招商，继续组织到珠三角发达地区招商，办好今年“肇庆金秋”经贸洽谈会。积极推进农业、文化、旅游、房地产、商贸物流等领域招商。在确保引进外资规模扩大、总量增加的基础上，调整优化招商引资策略和结构，重点引进投资规模大、技术含量高、产业链条长、资金密集型的项目，尤其是争取引进跨国公司、世界500强企业和其他知名企业，努力提高引进项目的经济效益和社会效益。切实加强已签约项目的投资落实和跟踪服务，提高项目履约率和资金到位率。鼓励引导现有外资企业达标达产和增资扩产，积极延伸产业链，迅速做大做强。继续实施“名牌带动”战略，扩大外贸出口，优化出口商品结构，支持具有自主知识产权和自有品牌的产品出口，鼓励高技术含量、高附加值的产品出口，提高外贸出口增长质量。加强口岸协调管理，加快“大通关”建设，提高口岸通关效率。高度重视民营经济，认真落实各项支持民营经济发展的配套政策，积极帮助中小企业解决融资难、用地难、招工难等问题。主动承接珠三角核心区民营资本和产业转移项目，鼓励支持民营企业参与国企改革重组，大力扶持本地民营企业发展壮大。

　　（三）加快建设社会主义新农村，促进县域经济协调发展。认真落实今年中央一号文精神，加强农村生产力建设，把促进农民增收作为中心任务，扩大农村基层民主，抓好农村各项改革，推进农村精神文明建设，促进农村和谐发展。加快农村工业化，做大做强山区林产化工、林浆纸一体化、矿产加工、水泥制造等资源型产业，培育县域特色优势产业集群。积极推进农村城镇化，重点抓好20个中心镇的规划建设。提高农业产业化水平，加快优势主导产业和产品基地建设，培育和扶持农业龙头企业，组织农产品加工和流通。加大农村水利、道路、自来水、电网、通信等基础设施建设投入，加强农业防疫体系建设，提高农业综合生产能力和抗击自然灾害能力。加强农村公共事业和教育、文化、卫生事业建设。抓好“三个一千工程”（千村生态文明工程、千村千干带头致富工程、千名党员帮万家工程），力争年内再建成500条生态文明村。保证财政支农资金高于上年，大力改善农村生产生活条件，办好8个镇、24条村的社会主义新农村建设示范点。

　　（四）积极组织落实重大项目，加快项目建设进程。全面动工建设广贺高速公路三水至怀集段，确保按计划2008年底建成通车。争取珠外环高速公路肇庆段、高新区至广州新机场快速干线、广梧高速公路封开连接线（含开南大桥）等年内开工建设。全面完成四清线四会至清远段改建工程，推进国道321线改造、镇通村公路硬底化等工程建设。加快肇庆新港建设和西江航道肇庆至封开界首段综合整治。做好广茂铁路复线、改线工程项目前期工作。规划建设华南国际医药开发基地等生产性项目。争取省液化天然气项目二期进入肇庆城区。加快景丰联围等一批重点水利工程建设。继续抓好正在谋划的重大项目的跟踪落实工作。

　　（五）以旅游业和物流业为重点，大力发展第三产业。深化旅游体制改革，增加旅游投入，加快旅游资源整合开发，推进千里旅游走廊建设。加强旅游项目宣传推介，打造“悠游肇庆岭南山水画廊”旅游品牌形象。培育现代物流业，积极引进国内外大型物流企业，支持福加德公司参与我市港口资源整合，建设港口物流基地。大力发展现代服务业，支持已引进的沃尔玛等大型商贸企业加快建设进度。抓好房地产业规划建设，引导房地产业健康发展。加强文化与经济、旅游、信息产业的融合，促进文化产业发展。

　　（六）深化国有企业改革，积极推进体制创新。加大国企改革攻坚力度，基本完成国有中小企业产权制度改革。把深化国企改革与加强技术改造、加强管理结合起来，积极引入战略投资者，鼓励社会各类资本参与国有企业改革重组。重点抓好风华集团、星湖科技、蓝带集团等市直国有骨干企业的改革发展，消除体制障碍，盘活壮大存量资产，加强内部管理，强化考核措施，力争市直工业生产效益有较大回升。加强国有资产监管营运，认真执行专项监管制度，抓紧制定国有资产收益管理办法等工作制度，完善国有资产监管机制，确保国有资产保值增值。深化行政审批制度改革，抓好第三轮行政审批事项的清理调整。深化财政管理体制改革，完善部门预算管理制度、国库集中支付制度、基建资金集中管理制度、财政重点项目绩效评价制度和政府投资项目预算约束制度，扩大政府采购范围，规范财政支出管理。加快要素市场建设，发展各类社会中介组织。落实“打假”工作责任制，维护市场经济秩序。建立完善中小企业信用担保体系，帮助支持中小企业畅通直接融资渠道。抓好资源节约，推进资源节约型社会建设。

　　（七）加大城市规划建设管理力度，强化生态环境保护。加快修编完善城市总体规划和专项规划。积极筹建贯通肇庆城区与南岸的西江新桥，促进“一江两岸”协调发展。继续抓好城#路网改造，加快端州七、八路建设，着手进行人民北路开通和黄塘路等一批城区道路改建。推进宋城墙修葺和周边环境整治。加快城东新区建设，促进肇庆城区向东拓展。加强城市综合管理，继续实施“美化、绿化、亮化”工程，迎接国家园林城市复检。加大生态环境保护建设力度,营造生态公益林体系，继续创建林业生态县。实施治污保洁工程，加强污染源预防和整治，加快城区垃圾场扩容工程、各县（市）生活污水处理厂和无害化垃圾场建设；抓好西江、星湖等重点流域和饮用水水源地保护，抓紧开辟城区第二饮用水源，确保市、县城区大气环境质量达标，巩固国家环保模范城市创建成果，推进环境友好型社会建设。

　　（八）认真做好财税、金融工作，确保财政收支平衡。大力培育税源，强化税收征管，提高财政收入增长质量。拓宽财政收入领域，搞好土地储备和经营，推进政府性资源收益统一管理，加大行政事业单位经营性资产清理力度，加强国有资本经营收益征收和管理，增加政府可支配财力。坚决控制会议费、接待费等一般性支出，优先保障科技教育、社会保障、公共卫生、民心工程等社会公共事业的投入。依法规范政府举债行为，建立健全地方政府债务监管偿还机制，有计划、有步骤地逐步消化政府债务，降低财政风险。加强金融生态环境建设，正确处理好支持地方经济增长和防范金融风险的关系，积极争取金融机构加大对地方经济社会发展的信贷支持力度。

　　（九）坚持以人为本，切实解决群众生产生活中的困难和问题。深入推进“十项民心工程”，重点改善农村生产、生活环境和解决医疗、教育、就业、社保等民生问题。通过省支持和市自筹投入1.2亿元、解决农村10万人饮水安全问题，计划投入1300万元扶持2498户农村贫困户危房改造，扶持培训并转移贫困劳动力3000人以上，对13条村、222户边远分散老区村庄进行搬迁补贴。开展减轻农民负担专项治理，不折不扣地把粮食直补、粮食最低收购价、农业机械购机补贴等优惠政策落到实处。深化并镇、并村、并校等各项配套改革。加快新型农村合作医疗制度建设，市级财政安排1100万元补助农村合作医疗。建立完善城乡贫困居民生活补助、灾害救助、医疗救助、教育救助、住房救助、法律援助、五保供养体系。继续投入2500万元，推进城区500套安居住房建设，确保年底前交付使用。加强医疗收费和药品价格监管，降低医疗成本。发展社会慈善事业，加强对流浪儿童的救助保护。推进社会保险扩面征缴，完善养老保险市级统筹。开展被征地农民基本养老保障试点，初步建立“双轨运行”的农村和城镇社会保险制度。推进城乡统筹就业，继续实施农村青年技能培训工程和创业培训富民工程，扩大劳务输出。健全劳动保障监察执法和劳动争议仲裁机构，切实维护劳动者合法权益。抓好工程款和农民工工资的清欠，争取年底前全面完成建设领域拖欠工程款清欠任务。

　　（十）积极推进科教兴市和文化名市建设，繁荣发展社会各项事业。加大科技投入力度，积极开展产学研联合，加强工程技术研究开发中心、专业镇、农业科技创新中心和农业科技示范镇建设。发展高新技术产业，抓好科技园区建设，完成市科技中心一期工程和院士活动基地建设。健全生产力促进中心等各类科技中介组织，筹划设立科技风险投资机构。高度重视人才工作，继续实施高层次人才“211工程”，强化人才队伍的专门培训和继续教育，抓好人才引进和培养，营造有利于人才发挥才干的环境和条件。加强科普工作。稳步推进专利和防震减灾、气象预测工作。切实把教育摆在优先发展的地位，大力改善农村义务教育办学条件，从2006年9月新学年开始全面落实免收农村义务教育阶段学杂费政策，市本级财政安排1391万元作为配套资金。认真解决端州区学位紧缺问题。加快普及高中阶段教育，办好重点高中，增加优质学位。加快建设“广东省肇庆职业技术教育培训基地”，积极发展高等教育，支持肇庆学院做好教育部的本科教学工作水平评估，抓好肇庆医专、技师学院、科技学院、工商学院新校区建设。积极推进文化名市建设，规划建设宋城墙、梅庵、阅江楼、崇禧塔等文化旅游景区。大力繁荣文化艺术，发展新闻出版、广播影视事业。深化文化体制综合改革，鼓励国有单位、社会组织和个人投资文化事业和文化产业，积极开展文化招商。开展丰富多彩的群众文化活动。加强精神文明建设，深入开展“爱国、守法、诚信、知礼”现代公民教育活动。建设学习型城市，举办首届“读书节”和“星湖学术论坛”，加强社区文化建设。实施共铸诚信工程，打造“诚信肇庆”。深入开展群众性精神文明创建活动，扎实推进创建全国文明城市工作。优化卫生医疗资源配置，适度发展民营医疗机构，加快市县两级疾控中心和市医疗紧急救援中心建设。改造33间乡镇卫生院，完善农村医疗预防保健网络，加强卫生支农，力争50%的乡镇实现农村初级卫生保健目标。抓好禽流感、非典、艾滋病等重大传染病防治。积极开展爱国卫生运动和无偿献血活动。大力发展中医中药事业。加强人口和计划生育工作，落实层级动态目标管理责任制，完成各项人口计划控制指标。围绕备战第十二届省运会，加强竞技体育工作，大力发展业余体育训练，广泛开展群众性体育活动。切实保护妇女、未成年人、老龄人和残疾人权益。大力推进殡葬改革。积极开展国防教育，推进国防动员信息化，加强民兵预备役和人防建设，深入开展“双拥”活动，扎实做好优抚安置工作，巩固和发展军政军民团结，争创全国“双拥模范市”。落实民族、宗教政策。加强外事侨务和对台工作，拓展对外交流领域。

　　（十一）切实维护社会稳定和谐，提升文明法治水平。加强民主法治工作。自觉接受人民代表大会及其常委会的监督，坚持向人大及其常委会报告工作和向政协通报情况制度，认真执行人大及其常委会的决议，及时办理人大议案、人民代表建议和政协的建议、提案。自觉接受人民政协、民主党派和无党派人士的民主监督，听取他们的建议和意见。加强城乡基层民主政治建设，推进社区居（村）委会直选，推动企事业单位民主管理。完善面向社会的新闻发布、社会公示与听证制度。扎实开展“五五”普法教育，增强全民法制观念。完善法律援助和司法救助制度。坚持依法征地，切实保护被征地农民的合法权益。加强社会管理，推进平安和谐社区建设，完善社区服务功能。开展创建和谐企业、和谐村镇活动。发挥工青妇及工商联等人民团体在社会管理中的作用，培育、发展和规范社团、行业组织。完善安全生产监管机制，落实安全生产责任制，增加对安全生产设施和防护救援装备的投入，强化对食品、药品、危险化学品、矿山、地质灾害和交通安全等的监管，遏制重特大事故的发生。加强维稳及社会治安综合治理工作。建立和完善政府应急工作机制，提高对突发公共事件的综合应急管理和紧急处置能力。健全各级调解组织网络，畅通诉求渠道，积极预防和妥善处置基层矛盾纠纷以及群体性事件。认真研究解决社会矛盾，做好信访工作，解决信访突出问题，完善化解社会矛盾的排查、预防、包案、联动机制。加强城市社区警务建设，健全社区治安防范网络。推进农村联防网络建设，增强农村治安防控能力。依法惩处各种严重刑事犯罪和经济犯罪，集中力量解决社会治安突出问题，坚决扫除“黄、赌、毒”和非法传销，保障人民群众生命财产安全。

　　(十二)加强政府自身建设，努力提高行政效能。按照省委、市委关于开展以“三个走在前面”为主题的排头兵实践活动的部署和要求，增强工作紧迫感和责任感，抓好各项工作的落实，着力建设为民、高效、廉洁、务实、有为政府。继续贯彻实施国务院《全面推进依法行政实施纲要》、《行政许可法》和《公务员法》，开展行政执法职权核准界定公告工作，推进依法行政。完善政务公开，积极推行公用事业单位办事公开制度。加强政府责任体系建设，落实政府行政首长问责制，完善工作目标责任考核制度。切实转变政府工作作风，认真解决审批环节中的各种问题，加强行政服务中心建设，提高行政效能。深入开展市直机关作风评议活动，切实解决好作风不实、办事拖拉和滥用行政权力等问题，提高服务效率。注意勤俭节约，反对铺张浪费。全面落实廉政建设责任制，贯彻落实《肇庆市建立健全教育、制度、监督并重的惩治和预防腐败体系实施办法》，发挥审计监督和行政监察职能，抓好治理商业贿赂专项工作，坚决查处违法违纪案件，坚决纠正损害群众利益的不正之风。

　　各位代表，我们要以强烈的政治责任感和历史使命感，在中共肇庆市委的正确领导下，全面贯彻落实市委九届五次全会精神，团结奋进，开拓创新，扎实工作，努力完成今年的各项任务，为“十一五”规划开好局，为建设繁荣活力、文明法治、和谐安康肇庆而努力奋斗！
